# Enhancing Human Immunodeficiency Virus-Specific CD8^+^ T Cell Responses with Heteroclitic Peptides

**DOI:** 10.3389/fimmu.2015.00377

**Published:** 2015-07-23

**Authors:** Adeolu Oyemade Adegoke, Michael David Grant

**Affiliations:** ^1^Immunology and Infectious Diseases Program, Division of BioMedical Sciences, Faculty of Medicine, Memorial University of Newfoundland, St. John’s, NL, Canada

**Keywords:** heteroclitic peptide, CD8^+^ T cell, therapeutic vaccines, HIV, epitope

## Abstract

Human immunodeficiency virus (HIV)-specific CD8^+^ T cells play a critical role in containing HIV replication and delaying disease progression. However, HIV-specific CD8^+^ T cells become progressively more “exhausted” as chronic HIV infection proceeds. Symptoms of T cell exhaustion range from expression of inhibitory receptors and selective loss of cytokine production capacity through reduced proliferative potential, impaired differentiation into effector cells and increased susceptibility to apoptosis. While effective combination antiretroviral therapy (cART) durably reduces HIV viremia to undetectable levels, this alone does not restore the full pluripotency of HIV-specific CD8^+^ T cells. In a number of studies, a subset of peptide epitope variants categorized as heteroclitic, restimulated more potent cellular immune responses *in vitro* than did the native, immunizing peptides themselves. This property of heteroclitic peptides has been exploited in experimental cancer and chronic viral infection models to promote clearance of transformed cells and persistent viruses. In this review, we consider the possibility that heteroclitic peptides could improve the efficacy of therapeutic vaccines as part of HIV immunotherapy or eradication strategies. We review literature on heteroclitic peptides and illustrate their potential to beneficially modulate the nature of HIV-specific T cell responses toward those found in the small minority of HIV-infected, aviremic cART-naïve persons termed elite controllers or long-term non-progressors. Our review suggests that the efficacy of HIV vaccines could be improved by identification, testing, and incorporation of heteroclitic variants of native HIV peptide epitopes.

## Introduction

Sequence variants of native or reference peptide are commonly referred to as analog peptides, mimotopes, altered peptide ligands (APL), or variant peptides. Heteroclitic peptides are a subset of these sequence variants that stimulate stronger T cell responses than the native epitope or reference sequence used for primary immunization (1, 2). Amino acid (aa) substitutions made in the peptide sequence of heteroclitic peptides presumably increase peptide antigenicity and immunogenicity by enhancing peptide-binding affinity for human histocompatibility-linked leukocyte antigens (HLA) and/or improving T cell receptor (TCR) recognition of the bound peptide (3–5). Given that thymic T cell positive selection is ultimately based on moderate affinity TCR binding to self-peptides presented on cortical epithelial cells, the concept of reference or native epitopes in an immune response arising against a foreign pathogen is purely contextual, from a T cell repertoire perspective. The reactivity of any individual T cell clone is selective not for one particular peptide, but for an entire series of related peptides that elevate TCR interaction avidity relative to that manifest through interaction with the original self-peptide. Although it can be addressed experimentally with sequence variants, where any particular reference, native or wild-type (WT) foreign peptide recognized by host T cells sits among the virtual series of peptides enabled for recognition through the original selection by a self-peptide, is unknown. Thus, there is a clear theoretical rationale suggesting that sequence variants can readily be constructed to mediate enhanced immune recognition compared to empirically defined reference, native or WT foreign peptides.

The finding that T cells from mice immunized with pigeon cytochrome c made stronger proliferative responses *in vitro* against tobacco hornworm moth cytochrome c than against pigeon cytochrome c itself was the first documented example of a heteroclitic peptide stimulating an enhanced immune response (6). Subsequent findings in various other experimental systems reiterated these seminal findings of Solinger et al. that certain peptide variants can restimulate more potent immune responses than the native, WT, reference or index peptide epitopes the immune system was actually exposed to (4, 7–14). In this review, we will focus on the possible utility of heteroclitic peptides for immunotherapy of human immunodeficiency virus (HIV) infection.

## Heteroclitic Peptides Augment and Modulate T Cell Cytokine Production

Cytokines play a significant role in orchestrating T cell development, differentiation, effector functions, and survival (15). Several experimental models have shown that heteroclitic peptides can enhance T cell cytokine responses beyond those stimulated by native peptides (4, 16–18). In 1997, Tao et al. demonstrated that heteroclitic peptide stimulation of CD4^+^ T cells *in vitro* significantly increased production of both interleukin-4 (IL-4) and interferon-gamma (IFN-γ), compared to stimulation with WT peptides (19). Salazar et al. found that a heteroclitic variant (CAP1-6D) of an immunodominant human carcinoembryonic antigen (CEA)-specific CD8^+^ T cell epitope (CAP1; YLSGANLNL) triggered a 1,000-fold increase in granulocyte-macrophage colony-stimulating factor (GM-CSF) and IFN-γ over the levels induced by CAP1 (4). In chronic infections such as HIV, development of antigen-specific T cell dysfunction, or exhaustion, is often reflected in sequential loss of cytokine production capacity, first with reduced ability to produce IL-2 followed by tumor necrosis factor-alpha (TNF-α) and lastly, loss of IFN-γ production (20–22). Variants of immunodominant HIV Gag and Nef epitopes tested *in vitro* by Gladney et al. enhanced IFN-γ and/or interleukin-2 (IL-2) production by HIV-specific CD8^+^ T cells compared to stimulation with the reference peptides, demonstrating heteroclitic properties in the context of cytokine modulation (23). In some cases, the effects were purely quantitative with an increased number of cells producing IFN-γ, IL-2, or both, but there were also cases where variant peptides selectively skewed cytokine production toward IL-2 (23). Thus, in settings where chronic infection leads to T cell exhaustion, the heteroclitic capacity of variant peptides may enhance nominal T cell functions or even reconstitute T cell functions no longer stimulated by native peptide epitopes. In follow-up experiments, additional heteroclitic-HIV peptides that stimulated larger cytokine responses, as reported by Gladney et al., were identified and then tested for their ability to increase T cell proliferation and reduce phenotypic evidence of exhaustion relative to *in vitro* stimulation with native peptides (24).

## Heteroclitic Peptides Enhance T Cell Proliferation

Failure to contain tumor growth is partly attributed to suboptimal proliferation of tumor-specific T cells responding against poorly immunogenic tumor-associated antigens (TAA) (25). To circumvent this issue, heteroclitic peptide variants of TAA were used to expand naïve TAA-reactive T cells and generate more effective antitumor T cell responses (26–28). In certain cases, more powerful adjuvants can also be used to overcome the relative immune tolerance to TAA or the relatively weak binding of self-peptides to HLA molecules (29). Another example of heteroclitic peptides enhancing T cell proliferation was *in vitro* expansion of T cells using an analog of an autoantigenic peptide 139–151 (HSLGKWLGHPDKF) from myelin proteolipid protein (PLP) in which tryptophan (W) was replaced by glutamine (Q) at position 144 (30). While the first case leads to an enhanced anti-tumor response and the second promotes autoimmunity, the same principle is illustrated in that weak T cell responses, due in part to self-tolerance, can be invigorated using heteroclitic stimulation. In chronic HIV infection, reduced proliferation of HIV-specific T cells with progressive infection reflects T cell exhaustion, which while distinct from anergy due to self-tolerance may be modulated through the same approach. We found that heteroclitic HIV peptide stimulation increased HIV-specific CD8^+^ T cell proliferation by at least 20%, and up to 1100% relative to reference peptide stimulation for nearly 50% of the heteroclitic/reference peptide pairs we compared (24).

## Heteroclitic Peptides Activate T Cells with High Avidity

Functional avidity is a measure of the amount of antigenic peptide required to successfully activate or trigger the effector functions of activated T cells (31, 32). Those T cells that respond efficiently at low doses of antigen are called high-avidity T cells (33). Several studies indicate a strong correlation between pMHC:TCR stability and functional T cell avidity (34–36). The enhanced anti-tumor cell-mediated immunity associated with loss of tolerance to TAA in studies using heteroclitic TAA peptides was due to increased pMHC:TCR stability of the heteroclitic peptides, reflected in higher avidity and longer interaction times (8, 13, 28, 37). Similar effects were observed when the *Mycoplasma penetrans* HF-2 (MPHF2) permease protein-derived peptide (IYIFAACL) was used to stimulate melanoma antigen gene (MAGE)-A6-reactive CD4^+^ T cells (38). The functional avidity of MAGE-A6-reactive CD4^+^ T cells stimulated with MPHF2 peptide was approximately 1000 times greater than that of MAGE-A6-specific CD4^+^ T cells stimulated with the WT MAGE-A6 peptide (IGH*VYIFATCL*GLSYD) (38). While the absolute number of HIV-specific CD8^+^ T cells recruited during primary HIV infection is undoubtedly important, HIV-specific CD8^+^ cells with high-avidity for HIV peptide epitopes are key to early HIV suppression (39–42). In contrast to the well-documented examples of heteroclitic TAA peptides inducing high-avidity T cells, there has been limited study of high-avidity T cell generation using heteroclitic peptides in the context of HIV infection. Stimulation with heteroclitic variants of an HIV-reverse transcriptase (RT) peptide (residues 309–317) significantly increased cytotoxic T lymphocyte (CTL) responses against the native pol peptide in all donors tested in comparison to stimulation with the native pol peptide (43, 44). A threefold increase in levels of the HLA-A*0201-peptide complex on the surface of antigen-presenting cells (APC) pulsed with the heteroclitic variants was thought to underlie the enhanced CTL responses (43, 44). Another study suggested that HIV protease (PR) peptide 76–84 (LVGPTPVNI), acting as a heteroclitic variant of IFN-γ-inducible protein 30 (IP-30) signal peptide −11 to −3 (LLDVPTAAV), activates autoreactive T cells in a subset of HIV-1-infected individuals expressing HLA-A2 (45, 46). Therefore, exposure to heteroclitic peptides can potentially cut both ways with promotion of autoimmunity through the activation of self-reactive T cells an equally possible outcome as enhancement of T cell responses against foreign or tumor antigens.

## Heteroclitic Peptides Enhance CTL Activity

Recognition and killing of infected cells is an important immunological effector function against HIV and other viruses and the critical importance of HIV-specific CTL in controlling viremia is well documented (35, 36). The ability to upregulate production of cytotoxins (perforin and granzymes) and to degranulate in a manner releasing them toward target cells is associated with viral control in long-term non-progressors (LTNP) and, conversely, is usually impaired in rapid progressors (RP) (47–49). Cytolysis of target cells by CTL can also be mediated through the Fas-Fas ligand (FasL) pathway, but perforin–granzyme release through degranulation is thought to be the predominant mode of killing of infected target cells by HIV-specific CTL (47). Several analogs of the HIV (RT) peptide ILKEPVHGV were tested for their ability to enhance HIV pol-specific CTL activity. Stimulation of peripheral blood mononuclear cells from HIV-infected individuals *in vitro* with I1→Y and I1→F variant peptides generated significantly higher WT pol-specific CTL activity compared to stimulation with the WT pol peptide (ILKEPVHGV) (43, 44). Likewise, for a series of subjects with chronic lymphocytic leukemia (CLL), CTL that were generated by stimulation with the heteroclitic immunoglobulin (Ig) variable gene framework region (FR)-derived peptide (Q*L*PGKGLEWV) had enhanced cytotoxicity against APC pulsed with the native peptide (FR-18; QAPGKGLEWV) and killed CLL cells, but not unpulsed, CD40-activated APC (13, 50, 51). In another example, CTL raised by stimulation with a heteroclitic FR-9 peptide (*K*LFLQMNSL) effectively lysed APC pulsed with the heteroclitic peptide and CLL cells displaying the native epitope (FR-9; TLFLQMNSL), while CTL generated by stimulation with the native peptide FR-9 failed to lyse either APC stimulated with the native peptide or FR-9-positive CLL cells (51). In a separate study, heteroclitic-variants of CD33, a cell surface glycoprotein restricted to myeloid lineage cells, effectively generated acute myeloid leukemia (AML)-specific CTL, that did not lyse or inhibit the proliferation of normal CD34^+^ progenitor cells (11, 12). These findings indicate that heteroclitic peptide stimulation often can generate substantially more effective CTL responses than the native epitopes. In certain situations, they can even activate autoreactive T cells in such a way that allows selective killing of infected or transformed cells over normal healthy cells. Induction of stronger, more durable, higher avidity T cell responses could be a powerful aspect of preventive and therapeutic HIV vaccines incorporating heteroclitic peptides.

## Heteroclitic Peptides Induce Less Evidence of T Cell Exhaustion

In HIV infection, chronic expression of programed death-1 (PD-1) molecules on HIV-specific T cells is a signature of functional T cell impairment that is clearly associated with disease progression (52–55). The HIV-specific CD8^+^ T cells of elite controllers (EC) and LTNP express significantly less PD-1 than those of normal or fast progressors (56). Several studies aimed at improving exhausted T cell responses used monoclonal antibodies to block either PD-1 or its ligands or introduced exogenous costimulatory molecules such as 4-1BB. In each case, the intervention improved the effector functions of responding T cells to some extent (53, 57). However, native HIV-peptide epitopes were used to stimulate HIV-specific CD8^+^ T cells in these instances, suggesting that even more benefit might be derived using heteroclitic peptides. A recent study demonstrated that heteroclitic variants of native HIV-peptide epitopes can improve the character as well as the magnitude of HIV-specific CD8^+^ T cell responses relative to responses induced by reference epitopes, even without introducing exogenous costimulatory molecules or blocking inhibitory receptors. In approximately 50% of cases tested, the fraction of proliferating HIV-specific CD8^+^ T cells expressing PD-1 was significantly reduced (by 15–88%) following stimulation with heteroclitic peptides in comparison to stimulation with reference peptides (24). While the effect of heteroclitic peptide stimulation on expression of T cell exhaustion markers was not studied in the other experimental systems cited, it is possible that the improved T cell responses were at least partially related to reduced exhaustion of the stimulated T cells.

**Figure 1:**
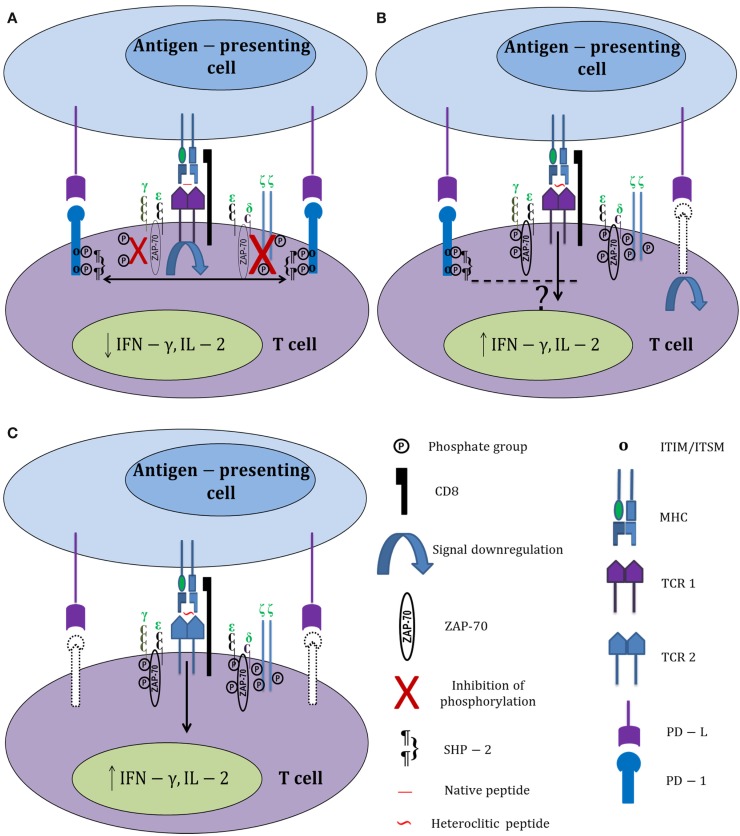
**Differential activation of HIV-specific CD8^+^ T cells by native and heteroclitic peptides**. **(A)** HIV-specific CD8^+^ T cell stimulation with HIV-1 native peptide epitopes increases PD-1 expression. PD-1 ligation with its ligand (PD-L) causes immediate phosphorylation of the immunoreceptor tyrosine-based inhibitory motifs (ITIM) and immunoreceptor tyrosine-based switch motifs (ITSM). Recruitment of SHP-2 to the tyrosine residues associated with PD-1 dephosphorylates TCR ζ chains, downregulates TCR signaling, and prevents the phosphorylation of ZAP-70. **(B)** Heteroclitic peptide stimulation increases tyrosine phosphorylation of ZAP-70 and TCR ζ chains, and also downregulates PD-1 expression on responding T cells (downregulated PD-1 is shown in dotted lines). Signals generated by heteroclitic peptide stimulation through the TCR somehow bypass the PD-1 signaling pathway; thereby improving T cell responses. **(C)** Heteroclitic peptide stimulation selectively activates a different subset of HIV-specific CD8^+^ T cells that express less PD-1 than the CD8^+^ T cells responding to the native peptide.

Surprisingly, there was no significant correlation between reduced PD-1 expression and enhanced proliferation in experiments carried out with heteroclitic peptides. This could mean either that heteroclitic peptides select for distinct heterogeneous subsets of HIV-specific CD8^+^ T cells or that activation signals generated by heteroclitic peptide stimulation can somehow bypass the PD-1 signaling pathway, thereby allowing enhanced proliferation even of PD-1^hi^ CD8^+^ T cells (Figure 1). Normally, inhibitory signals delivered by PD-1 downregulate TCR signaling through direct dephosphorylation of intracellular signaling intermediates. The phosphatases associated with PD-1 (SHP-1 and SHP-2) dephosphorylate CD3ζ and prevent phosphorylation of ZAP-70 and PKCθ (58). A possible explanation for the negligible effects of PD-1 signaling on proliferating HIV-specific CD8^+^ T cells responding to heteroclitic peptide stimulation could be increased tyrosine phosphorylation of ZAP-70 and TCR ζ chains in CTL stimulated with heteroclitic peptides relative to native peptide-stimulated CTL. In fact, this was reported in the study by Salazar et al. and supports the possibility that heteroclitic peptides can potentially bypass the pathways effecting exhaustion when stimulation with reference peptides occurs (4). A more recent publication demonstrated differential effects on the PD-1 expression of HIV-specific CD8^+^ T cells in relation to their TCR clonotype as well as epitope specificity and *in vivo* history of antigenic exposure (59). Thus, a variety of intrinsic and extrinsic factors can modulate translation of T cell stimulation by even closely related peptides into a range of potential outcomes.

## Heteroclitic Peptides for “Kick and Kill” Approaches to Purge the HIV Reservoir

Despite the spectacular success of combination antiretroviral therapy (cART) in treating HIV infection, it remains impossible to cure HIV infection in all but extremely rare cases. Cure could either be sterilizing, with eradication of all HIV-infected cells and infectious viral particles, or functional, with long-term suppression of HIV replication in the absence of cART (60). The “Berlin patient,” who was transplanted with allogeneic stem cells from a CCR5 delta32-homozygous donor, is regarded as the only documented case of sterilizing cure (61–63). Other reported cases of HIV cure upon cART cessation such as the VISCONTI cohort and the “Mississippi baby” represented functional cures with unknown, and as we now know, questionable durability (64–68). Viral rebound in individuals taken off treatment or once classified as “functionally cured” is attributed to the long-lived HIV reservoir persisting in latently infected cells (69). An approach to HIV cure termed the “kick and kill” strategy has been developed to overcome the challenges posed by this long-lived HIV reservoir. This approach aims to reactivate latent viruses with histone deacetylase inhibitors and other agents (the “kick”) and then expose the reactivated viruses and infected cells to HIV-specific immunity and/or cART (the “kill”) (70). Concerns have been raised as to the capacity of endogenous CTL to recognize and mediate their effector functions with the efficacy required to extinguish reactivated HIV (71). Sung et al. showed that *in vitro* restimulation and expansion of endogenous anti-HIV CTL produced effector cells targeting the latent reservoir (72). Either *in vivo* or *in vitro* restimulation of anti-HIV CTL with defined heteroclitic peptides in concert with the kick approach to reactivate latent virus from the reservoir could enhance the capacity of anti-HIV CTL to effectively target reactivated virus.

The potential for heteroclitic peptides to generate higher avidity cross-reactive CTL to help purge escape variants that significantly contribute to residual viremia was previously documented in murine hepatitis virus (MHV) infection (73). Two MHV strain JHM (JHMV)-derived CTL epitopes, S510 (CSLWNGPHL) and S598 (RCQIFANI), were shown to induce very high- and low-magnitude T cell responses, respectively, that select for escape mutants during chronic MHV-infection in C57BL/6 (B6) mice. The immunodominant epitope, S510, generates high-avidity CTL responses; whereas an approximately 100-fold lower avidity T cell response is generated against the subdominant epitope, S598. To improve CD8^+^ T cell responses against S598, Butler et al. substituted a tyrosine residue for glutamine at position 3 of the S598 peptide sequence (Q600Y; RCYIFANI). Approximately, 100-fold more native S598 peptide is required to reach the response level induced by the heteroclitic Q600Y peptide. Immunization with Q600Y effectively protected against disease progression in the context of both S598 and S510 CTL escape mutations, and enhanced clearance of MHV (74). Thus, heteroclitic peptides could be effective against CTL escape in chronic HIV infection and also be beneficial in the “kick and kill” approach to HIV cure.

## Heteroclitic Peptides and HIV Immunotherapy

Unlike prophylactic vaccines, which provide uninfected persons protection against invading pathogens, therapeutic (treatment) vaccines are designed to modulate the ongoing immune response in unresolved infections or other chronic conditions. Therapeutic vaccines are generally engineered to induce cell-mediated, rather than humoral immunity by enhancing existing or generating new immune responses toward chronic pathogens or tumor antigens. While most HIV-infected individuals have strong CTL responses in the primary phase of infection, chronic infection ensues at least in part due to the ability of the virus to escape effective recognition by HIV-specific CD8^+^ T cells (75). Thus, viral persistence, reflecting inefficient recognition of HIV escape mutants, precedes the functional impairment of HIV-specific T cells (22). Although cART has dramatically reduced morbidity and mortality among individuals with chronic HIV infection, HIV-specific CD8^+^ T cell dysfunction persists through successful ART treatment, leaving the immune system in a compromised state (76, 77). In addition, the high cost, pill burden, requirement for strict adherence, and side effects are all drawbacks that limit universal application of cART to contain the HIV epidemic. The economic burden imposed by the HIV epidemic in both the developing and developed world has already hindered growth. For economic reasons alone, there is an urgent need to explore alternatives to cART, such as immune-based strategies that can reduce the requirement for life-time cART and ease associated physiological and economic burdens.

Potentially heteroclitic variants were previously generated using HLA-A2 and A3-restricted tumor-associated peptides (9mers and 10mers), an HLA-A2-restricted hepatitis B virus (HBV) peptide, Pol.455 and HIV Pol.476 (both 9mers). Regardless of their length, variant peptides with heteroclitic activity all had either conservative or semiconservative aa substitutions at positions 3, 5, or 7 and decreased the amount of peptide required to trigger T cell responses by up to 107-fold (37). Inferred 3D structures from X-ray crystallography of HLA-A2 and A3 pMHC:TCR complexes revealed that the side chains of aa at positions 3, 5, and 7 of these peptides bound to the MHC molecules interact directly with complementarity determining region 3 (CDR3) of the TCR α and β chains (78, 79). A table of aa similarity scores used in this study by Sette et al. was derived by averaging the rank coefficient score for tolerability of point mutations within a protein as estimated by the Dayhoff point accepted mutation (PAM) matrix 250, hydrophobicity calculated from the average of Kyte/Doolittle and Fauchere/Pliska scales estimating hydrophobicity and hydrophilicity, respectively, and aa side chain volume (measured by H_2_O displacement) for each aa pair (37). It was later shown that heteroclitic variants of HIV native peptide epitopes can be generated in a manner similar to that illustrated by Sette et al. using the same table to select conservative and semi-conservative aa substitutions at positions 3, 5, and 7 of native peptide epitopes (23).

## Design and Testing of Heteroclitic Peptide Vaccines for HIV

Potential barriers to developing generalized heteroclitic peptides for therapeutic vaccination against HIV include inherent individual diversity in HLA class I genotypes and TCR repertoires plus differential immunological responsiveness reflecting the variable impact of HIV infection itself. Our experience has been that HIV-infected individuals responding against the same WT peptide often do not demonstrate heteroclitic responses against the same variant peptides (23). Thus, heteroclitic peptide vaccines would need to be individually designed, at least initially. While not an optimal approach to vaccination, the overall investment is not prohibitive compared to drug resistance and viral tropism testing or when considered in terms of the potential to reduce antiretroviral dependence or contribute to HIV cure. Individuals would require mapping of their HIV-specific CD8^+^ T cell responses by standard methods first with available overlapping peptide pools and subsequently with deconvoluting matrices (23). Epitopes would be selected for further investigation from amongst those identified based on immunodominance and polyfunctionality of the responding T cells in terms of their cytokine production. The semi-systematic approach to generating candidate heteroclitic peptides by making conservative and semi-conservative aa substitutions at positions 3, 5, and 7 can probably be improved using population-based HIV sequence data sorted by HLA genotype. Analysis of such data indicates sites of immune selection with an estimable hierarchy and identifies putative adapted (escape) mutations and, more importantly, non-adapted or excluded aa at particular sites within CTL epitopes that are likely excluded due to enhanced immunogenicity of that sequence (80). Although it seems counterintuitive, it may be exactly these sequences selectively avoided by HIV in the context of certain HLA molecules, rather than broadly conserved sequences, that are the most effective immunogens against HIV. Large series of variant peptides for those epitopes most common in the population could be synthesized and tested *in vitro* for heteroclitic properties. The ideal heteroclitic peptide should activate a higher fraction of CD8^+^ T cells, stimulate a more polyfunctional response, increase CD8^+^ T cell proliferation, and drive differentiation of fully functional effector cells with equal or superior TCR recognition of the native peptides and existing variants. Individual *in vitro* testing for heteroclitic function reduces the risk that administered peptides will have partial agonist or antagonist functions that actually impair the CD8^+^ T cell response against HIV *in vivo*. Higher frequency *in vitro* responses do not necessarily translate into more effective responses *in vivo*. Vaccination with a heteroclitic peptide derivative (ELAGIGILTV) of Melan-A (EAAGIGILTV), which had higher affinity to HLA, induced twice the frequency of peptide-reactive T cells as vaccination with the natural peptide. However, analysis of responding T cells at the clonal level indicated a marked preference for recognition of the heteroclitic peptide over the natural peptide with recognition at lower peptide concentrations due to the increased affinity for HLA (28). Thus, TCR recognition efficacy may be overestimated in terms of the ability to mediate effector functions against target cells presenting the natural peptide. Choosing heteroclitic peptides with aa modifications at TCR interaction sites not affecting HLA-binding affinity may allow for more unbiased comparison of TCR recognition efficiency *in vitro*. The risk of activating autoreactive T cells must be measured against the potential benefits of heteroclitic vaccination in HIV infection. It is impossible to comprehensively test for autoreactivity against all self-antigens presented to CD8^+^ T cells using cells available from peripheral blood, but autoreactivity could also be tested against broad panels of cell lines expressing the same HLA class I antigen that presents the heteroclitic peptide variants identified. If more than one suitable heteroclitic peptide were identified, they could be administered individually, together or as a long synthetic peptide by subcutaneous injection with an approved adjuvant. Subjects would be monitored for an enhanced CD8^+^ T cell response against HIV following vaccination.

## Conclusion

The purpose of this review was to highlight the potential advantages of incorporating heteroclitic peptides into therapeutic vaccines, in particular into therapeutic HIV vaccines. Heteroclitic peptide stimulation has been shown to produce beneficial immune responses in a number of chronic infections and disease models (Table 1). Changes as subtle as a single conservative aa substitution within the native peptide epitope sequence can improve T cell responses in the form of enhanced cytokine production and increased proliferation. This can also produce higher-avidity T cell responses, more effective CTL killing, protection against CTL escape, elimination of chronic infection, specific abrogation of self-tolerance that elicits anti-tumor immunity, and prevention or reversal of T cell exhaustion. As with other peptide-based vaccines, heteroclitic peptide immunotherapy poses no risk of genetic integration or recombination, as with DNA vaccines, and no risk of developing virulent reassortants or of reversion to virulent (WT) viruses as with attenuated virus vaccines. Hence, administration of heteroclitic peptides is an exceptionally safe, potentially effective mode of vaccination. Heteroclitic peptide-based vaccines could be commercially produced on a large scale at low cost, affording those poor nations most plagued by the HIV epidemic the potential benefits of immunotherapy. Heteroclitic peptide vaccine formulations can also be stored in lyophilized form, thereby avoiding the need for “cold-chain” maintenance during storage, transport, and distribution. Consequently, they can be made readily available to the most remote and intemperate locations. The many demonstrated practical and theoretical advantages of heteroclitic peptides suggest that more research on their inclusion into therapeutic HIV vaccines is warranted.

**Table 1:** **Native peptides and heteroclitic variants cited**.

Native peptide	Heteroclitic variant	Reference
Pigeon cytochrome c	Tobacco hornworm moth cytochrome c	(6)
CAP1; YLSGANLNL	CAP1-6D; YLSGA**D**LNL	(4)
PLP 139-151; HSLGKWLGHPDDF	PLP 139-151, W144Q; HSLGK**Q**LGHPDDF	(29)
MAGE-A6; IGHVYIFATCLGLSYD	MPFH2; **I**YIFA**A**CL	(37)
IP-30 -11 to -3; LLDVPTAAV	HIV PR 76-84; **LVG**PT**PVNI**	(44)
HIV RT 309-317; ILKEPVHGV	HIV RT 309-317, I1Y; **Y**LKEPVHGV	(42)
HIV RT 309-317; ILKEPVHGV	HIV RT 309-317, I1F; **F**LKEPVHGV	(42)
FR-18; QAPGKGLEWV	FR-18; Q**L**PGKGLEWV	(13)
FR-9; TLFLQMNSL	FR-9; **K**LFLQMNSL	(50)
S598; RCQIFANI	S598, Q600Y; RC**Y**IFANI	(72)
Melan-A EAAGIGILTV	Melan-A E**L**AGIGILTV	(28)

*Native peptides and heteroclitic variants cited as examples in the text are tabulated together with the corresponding reference. Although nomenclature used is not always standard, the peptide sequence is generally preceded by an abbreviation indicating the source and protein name followed by aa sequence coordinates within that protein, site, and identity of the relevant aa substitution in the heteroclitic variant and the peptide sequence. Altered amino acids within the heteroclitic variant are shown in bold text*.

## Conflict of Interest Statement

The authors declare that the research was conducted in the absence of any commercial or financial relationships that could be construed as a potential conflict of interest.
